# Dissociating Empathy From Perspective-Taking: Evidence From Intra- and Inter-Individual Differences Research

**DOI:** 10.3389/fpsyt.2019.00126

**Published:** 2019-03-14

**Authors:** Julia Stietz, Emanuel Jauk, Sören Krach, Philipp Kanske

**Affiliations:** ^1^Faculty of Psychology, Clinical Psychology and Behavioral Neuroscience, Technische Universität Dresden, Dresden, Germany; ^2^Social Neuroscience Lab, Department of Psychiatry and Psychotherapy, University of Lübeck, Lübeck, Germany; ^3^Research Group Social Stress and Family Health, Max Planck Institute for Human Cognitive and Brain Sciences, Leipzig, Germany

**Keywords:** empathy, perspective-taking, theory of mind, lifespan development, personality, mental disorders

## Abstract

Humans have the capacity to share others' emotions, be they positive or negative. Elicited by the observed or imagined emotion of another person, an observer develops a similar emotional state herself. This capacity, empathy, is one of the pillars of social understanding and interaction as it creates a representation of another's inner, mental state. Empathy needs to be dissociated from other social emotions and, crucially, also from cognitive mechanisms of understanding others, the ability to take others' perspective. Here, we describe the conceptual distinctions of these constructs and review behavioral and neural evidence that dissociates them. The main focus of the present review lies on the intraindividual changes in empathy and perspective-taking across the lifespan and on interindividual differences on subclinical and clinical levels. The data show that empathy and perspective-taking recruit distinct neural circuits and can be discerned already during early and throughout adult development. Both capacities also vary substantially between situations and people. Differences can be systematically related to situational characteristics as well as personality traits and mental disorders. The clear distinction of affect sharing from other social emotions like compassion and from cognitive perspective-taking, argues for a clear-cut terminology to describe these constructs. In our view, this speaks against using empathy as an umbrella term encompassing all affective and cognitive routes to understanding others. Unifying the way we speak about these phenomena will help to further research on their underlying mechanisms, psychopathological alterations, and plasticity in training and therapy.

## Empathy and Perspective-Taking

When confronted with someone else's emotions, people often spontaneously share that affective state–your grief can become my grief, your joy, my joy. Such a vicarious, isomorphic emotion in an observer of another person's emotions has been referred to as *empathy*, a term introduced by Vischer and Lipps as “Einfühlung” (German for “feeling into,” derived from the Greek empatheia) (1). In humans, empathy may even arise, when the other is not present, but thought of or imagined. Critically, however, it has been proposed to involve self-other distinction, that is, the awareness that another is the source of one's emotions, differentiating it from emotional contagion, where such an awareness is not present (2).

**Figure 1:**
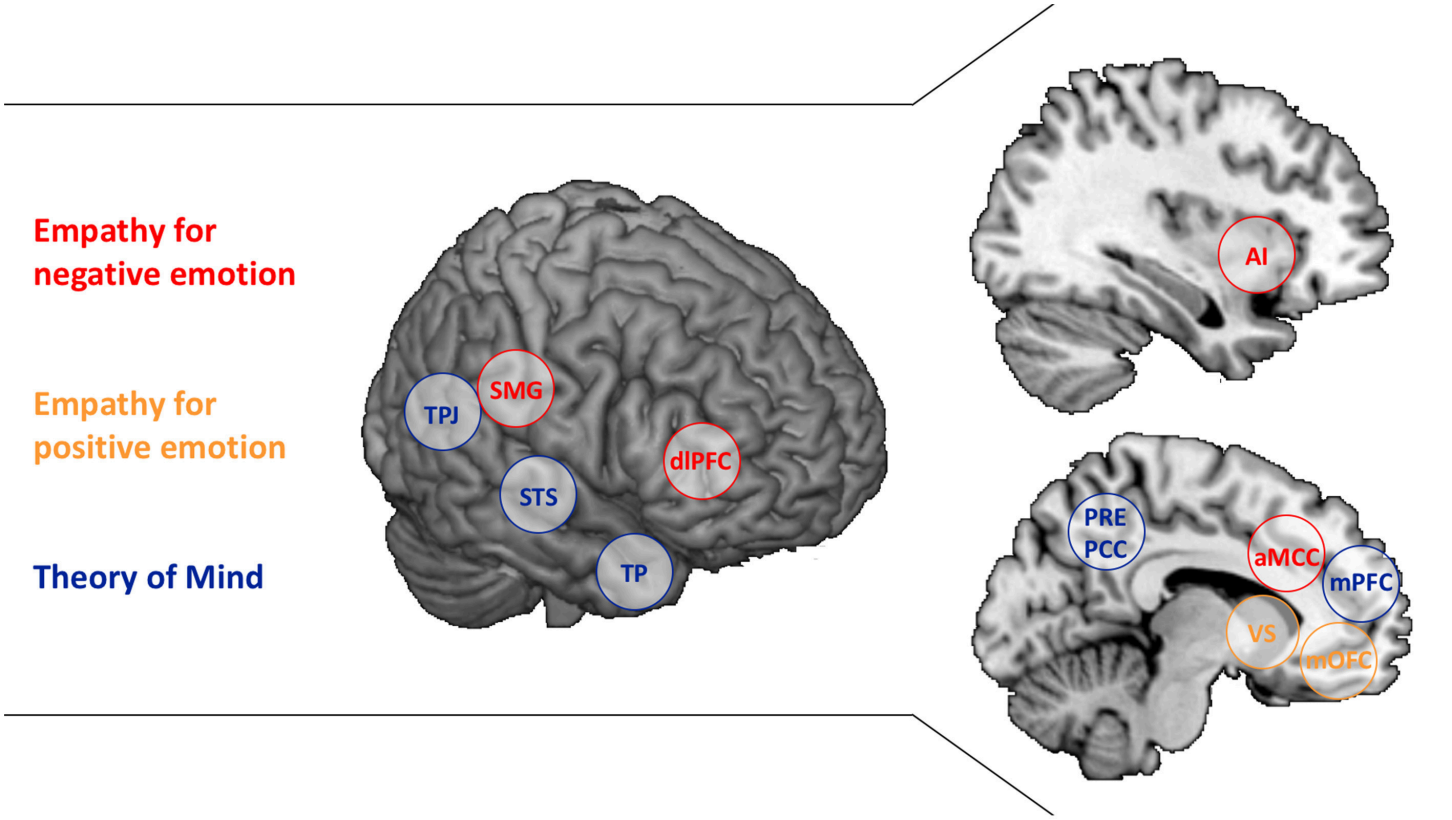
Brain regions associated with Empathy and Theory of Mind. The separable brain regions associated with Empathy for negative emotion (red), Empathy for positive emotion (yellow), and Theory of Mind (blue) are presented. AI, anterior insula; aMCC, anterior middle cingulate cortex; dlPFC, dorsolateral prefrontal cortex; mOFC, medial orbitofrontal cortex; mPFC, medial prefrontal cortex; PCC, posterior cingulate cortex; PCUN, Precuneus; SMG, supramarginal gyrus; STS, superior temporal sulcus; TP, temporal poles; TPJ, temporoparietal junction; VS, ventral striatum. SMG and dlPFC are listed as well as they have been associated with regulating empathic emotion (7).

Of course, empathic affect sharing is only one possible response to another person's emotion. Complementary affective states such as schadenfreude, envy or compassion occur as well, but the peculiarity of empathy is that it enables access to another's internal state by re-creating a representation of that state in the observer (3–6). Correspondingly, neuroscience research on empathy has not identified one single neural network associated with empathy, but rather the brain regions found to be active depend on what affective state is shared. While empathy for others' pain and negative affect activate the anterior insula and anterior midcingulate cortex (core nodes of the salience network), sharing others' joy and positive emotion yields activity in the ventral striatum and medial orbitofrontal cortex (Figure 1) (8–10). These activations seem to be relatively high-level, affective representations, as the specific patterns associated with one negative state, for instance, empathic pain, enable predictions of other negative states such as empathic disgust or unfairness (11). Furthermore, first-hand and empathic emotion experience—being stimulated painfully or watching someone else in pain—also lead to mutually predictive activation patterns in anterior insula and anterior cingulate cortex (12, 13). The observation of such “shared neural networks” has been interpreted as agreeing with simulation theory's account of how we understand others—we impersonate them and imitate their mental states (14).

Empathy, then, needs to be differentiated from an alternative route to understanding others. Theory theory, assumes abstract, propositional knowledge about others' behavior to underlie the understanding of the motives that drive others' behavior (15). This conceptualization corresponds to psychological and neuroscience research on *perspective-taking* or *Theory of Mind (ToM)*, the capacity to make inferences about and represent others' intentions, goals and motives (other terms include mentalizing and cognitive empathy) (16, 17). A classic test of ToM is false-belief understanding. If I can apprehend your incorrect view on a matter, while knowing the actual truth, the information conflicts and I must represent it in an abstract manner (18). Neuroscientific investigations of false-belief understanding have identified a network of brain regions to be involved, including the temporoparietal junction, precuneus/posterior cingulate cortex, medial prefrontal regions as well as the temporal poles and superior temporal sulcus (partially overlapping with the default mode network; Figure 1) (19, 20). While the main nodes of this network are also involved in other experimental paradigms assessing ToM, some regions within the overall network seem to be specific for particular ToM tasks (21, 22). Assuming a “constructivist view” on ToM (23), this may be due to different tasks drawing different component processes of ToM (24). Ecologically complex ToM tasks, in contrast, activate the entire network, possibly because all component processes are required (25, 26).

Thus, the abilities to empathically share others' affect and take their perspective can be well-differentiated conceptually and have more recently also been directly dissociated on a neural network level (for a summary see Table 1) (25). However, they may also interact and facilitate or impair one another in complex situations that require both functions simultaneously. For instance, Lamm et al. (8) meta-analytically contrasted cue-based and picture-based empathy for pain studies. When only presented with abstract cues of how painfully another person is stimulated, regions in the ToM-related neural network are activated, possibly reflecting the reasoning about the other's state, which then facilitates or enables empathic sharing of that state. In contrast, when the painful stimulation is directly displayed, ToM is not required to empathize and ToM-related neural activity is absent. Similarly, brain regions in inferior parietal and frontal cortex that have been associated with motor simulation [“mirror neuron system; ”(27)] can also trigger empathic responding, if an action needs to be understood for the affective consequences to become clear (28). Empathy and ToM can also show a different interactive pattern in highly emotional situations. Here, ToM performance has been found to be impaired, which is associated with an inhibitory influence of empathy-related anterior insula activation on ToM-related temporoparietal junction activation (29). This may reflect an adaptive response to highly salient situations requiring immediate action, but could also turn maladaptive as has been hypothesized with a stress-related mentalizing deficit in borderline personality disorder (30).

**Table 1:** Summary of the conceptual and empirical dissociation of empathy and perspective-taking.

**Empathy**	**Perspective-taking**
•Affective process	•Cognitive process
•Sharing another's emotional state	•Taking another's perspective
•Awareness that other is source of emotion	•Abstract representation of others' mental state
•Involved brain regions depend on emotional valence, largely overlaps with salience network	•Widespread network for information processing, core nodes overlap with default mode network
•Develops ontogenetically early, does not decline in old age	•Later ontogenetic development, declines in old age
•State/trait reductions mainly for motivational/habitual reasons	•State/trait reductions for motivational/habitual and cognitive reasons

Given the distinguishable neural networks enabling empathy and ToM, it is interesting to ask, if they share interdependent or distinct developmental trajectories over the lifespan, which we will discuss in the next section.

## Intraindividual Differences

### Lifespan Development

Speaking with the words of Hutman and Dapretto (31): “Determining the age at which infants display empathy depends in large part upon the way the construct is defined.” Defining empathy as above—as sharing others' emotions while being able to differentiate between oneself and the other—it could be argued that empathy emerges very early in life. Precursors of affect sharing, like emotional contagion, and indirect self-other distinction can already be observed in newborns, well before the emergence of verbal abilities (31–33). For instance, infants display greater and longer distress when confronted with the cry of another newborn compared to their own (34). During childhood, these capacities refine and become more explicit—they can be named and regulated (35–37). Thus, there is no clear age cut-off at which empathy is fully developed or not. Determining the age at which infants display empathy depends on the methods used to capture it—observational and physiological measures, adult-reports or self-reports—which vary highly in their validity and outcomes throughout development (38). With incremental development of its subcomponents and language abilities empathy becomes more apparent and easier to quantify in preschool children. It further develops during adolescence with increases from age 12 to 16 years (39). In sum, the emergence and development of empathy depends strongly on the definitions and methods used, but first signs of affect sharing are already present in newborns.

For ToM, numerous studies show that classical tests of false-belief understanding are not passed before the age of 4–5 years (40). However, when tested with non-traditional tasks, early preverbal ToM abilities such as mental state attribution and intentional communication seem to emerge already in infancy at 6–9 months of age, gradually developing further throughout the first years of life (18, 41–44). Setoh et al. (45) could further demonstrate that 2.5-year-olds are able to succeed in classic false belief tasks if overall processing demands are reduced by lowering inhibitory control and response-generation demands. This supports the view that ToM also develops incrementally, starting before the age of 4–5 years.

Taken together, empathy and ToM become well-measurable in preschool aged children with increasing abilities in language and executive function. Nevertheless, non-verbal precursors of both capacities are already observable in infancy, in newborns for empathy and from about 6 months on for ToM. Longitudinal studies testing both empathy and ToM jointly, which could yield the most profound evidence for independent trajectories throughout childhood, are still missing. Recently, a cross-sectional study examined empathy and ToM within a single group of children ranging from 3 to 5 years of age (46). Children had to pass a certain number of subtasks for empathy and ToM to be classified as having developed either ability. ToM seemed to emerge at 4 years and empathy at 5 years of age. Interestingly, a subgroup of kids, including 4-and 5-year olds, displayed empathy but not ToM. These results cannot yet answer if the development of empathy follows ToM or vice versa, but they hint at some independence in their developmental trajectories.

While numerous studies addressed the emergence of empathy and ToM in childhood, growing evidence also sheds light on their development in old age. In a recent cross-sectional study (47) younger and older adults performed a newly developed naturalistic task which measures both empathy and ToM within the same individuals [EmpaToM; (25)]. Older adults performed significantly worse than younger participants on the ToM questions whereas empathy was still preserved in older adults. These findings are in line with previous studies in younger and older adults, separately testing their abilities to empathize (48–50) and to take others' perspectives (51). The decline of ToM in older adults is a consistent finding across various ToM tasks regardless of stimulus modality or the specific form of ToM that is measured (52). For empathy, in contrast, no age-related changes or even increases with age have been reported (53–55). These findings depict independent developmental paths for empathy and ToM in old age.

Taken together, empathy and ToM evolve and decline independently during lifespan development. A number of factors have been found to influence this development, particularly in childhood, including preterm birth (56), child to parent attachment (37), language use of the parents (57, 58), mental disorders of the parents (59), the presence of older siblings (60, 61) and the specific culture a child grows up in (62, 63). Such influencing factors cause interindividual differences in empathy and ToM that could even reach into psychopathology and might be greatly informative regarding the relation of the capacities—a question we discuss in the following section.

### State Variability

While typically developed adults possess the capacity to empathize and take others' perspectives, there is still variation in the propensity to translate this capacity into actual behavior. Whether and to what extent we empathize with others or take their perspectives may depend on situational and relational variables as well as motivational factors (2). Empathic processes are generally more salient in situations in which we are confronted with negative rather than positive emotions [e.g., (64)]. We display stronger empathic reactions when interacting with those we are closely affiliated with (65), which points to a central role of empathy in human and non-human evolution (66, 67). This is supported by recent advances in understanding the role of oxytocin in both, empathy and attachment (68). Similarly, we tend to experience higher empathy toward ingroup others, and lower empathy toward outgroup others (69), even when group membership is experimentally varied (70). We typically experience low empathy in states of personal distress or depression (71), particularly due to an incapacity to inhibit own emotional states (72).

ToM is high in states in which we are motivated to understand others' mental states and intentions, which allows making predictions about their actions, and also to influence these actions (16). This can happen for altruistic or also egoistic motives. For instance, one might take another's perspective to be better able to help them, or also to effectively manipulate them. ToM can be low in states which may block the cognitive route to understanding others, such as alcohol intoxication (73), or also depression (74). Though reduced ToM in depression is frequently hypothesized to emerge from heightened egocentric focus, it is not fully understood whether alterations of ToM in depression, for example, are specific to social cognition, or might also be attributed to deficits in executive functioning (75). This highlights the necessity of controlling for general processing capacity in studies investigating individual differences in ToM.

Taken together, contextual factors substantially determine the extent to which we engage in empathy and ToM. Contextual factors may also guide whether we engage affective or cognitive routes to understanding others, which reflects in the respective neural activation (76).

## Interindividual Differences

Beyond transient variations in empathy and ToM, there are also interindividual difference variables that are reliably associated with dispositional variation.

At a most basic level, women score higher on self-report measures of empathy than men, which may be due to gender-role stereotypes (77) as gender differences are not clearly present in neural empathy responses [but seem to depend largely on context effects, (78)]. Among the Big Five personality traits, agreeableness is most consistently and strongly linked to variation in empathy [e.g., (79)], which has recently been substantiated by neuroimaging research (80). Agreeable individuals have a higher propensity to display empathic reactions, or conversely, empathy can be thought of as a low-level function that serves higher-order facets of agreeableness, such as altruism. Regarding lowered empathic responses, the “dark” personality traits narcissism, Machiavellianism and psychopathy (81) are commonly associated with reduced empathy [e.g., (82)]. These are tied together by interpersonal antagonism—the opposite of agreeableness—in terms of a self-focused and callous interpersonal style (83). Emotional contagion and empathy are typically lower in narcissism (82, 84, 85) and psychopathy (86, 87). Interestingly, empathic alterations in narcissism and psychopathy are not due to an incapacity to empathize, but rather due to motivational factors. Experimental evidence shows that narcissistic individuals experience regular levels of empathy when being instructed to put themselves into the perspective of a suffering person (88). Similarly, psychopathic individuals—viewed as similar, yet more severely disordered (89)—can indeed experience empathy. Psychopathic individuals show similar brain activation as controls in the anterior insula and anterior cingulate cortex, but only deliberately, not spontaneously (90). This confirms the notion of reduced propensity for empathic reactions, not reduced capacity in terms of general inability to share others' affect, in psychopathic individuals.

While the majority of individual differences research on empathy focuses on variables that are accompanied by lowered empathy, there are also examples in which empathy is hypothesized to be higher. For instance, clinical observations suggest the existence of “borderline empathy” in terms of surprisingly accurate emotional resonance in individuals with borderline personality disorder (91). The overall evidence on borderline empathy, however, is mixed (92), and some research indicates that the phenomenon might be conceptualized in terms of increased emotion recognition ability [e.g., (93)], which does not necessarily involve affective sharing.

Unlike empathy, variation in ToM is less clearly associated with sex [e.g., (94)], but similarly associated with the Big Five dimension of agreeableness; particularly when complex ToM measures are used (95). ToM is also not uniformly lowered in the Dark Triad traits [e.g., (96)]. A recent study found that only automatic ToM is lowered in psychopathy, whereas controlled ToM does not differ from controls (97). This points to a diminished propensity rather than capacity to take others' perspective, which highlights the motivational role of personality characteristics in ToM. Taking this idea one step further, there is even evidence for increased social cognition in individuals high on “dark” personality traits, which could enable antagonistic individuals to effectively deceive and manipulate others (98, 99).

Taken together, research on intra- and interindividual differences shows that there is substantial variation in affective and cognitive interpersonal functioning. Both can be selectively heightened or lowered, depending on state and trait characteristics. This corresponds to behavioral and neuroscience evidence showing that strong empathizers are not necessarily better mentalizers, and vice versa (29). Whether and to what extent we empathize and take others' perspectives depends substantially on situational and motivational variables, the latter of which reflect in personality traits. Altered social affect and cognition related to personality traits and disorders are likely more a matter of reduced propensity than capacity.

## Conclusion and Outlook

While the phenomena of affect sharing and perspective-taking may be relatively well-understood, there is considerable variation in the terminology used to describe them. The definition of empathy ranges from confining it to affect sharing [applied in the current review; (2)] to a very broad usage as an umbrella term. The latter view would merge (i) affect sharing, personal distress and emotional empathy as an emotional and (ii) mentalizing, perspective-taking and ToM as a cognitive component of empathy (100, 101). Here, we reviewed evidence that dissociates these functions, with differential neural networks related to empathy and ToM (Figure 1). Lifespan developmental research further indicates independent trajectories—the affective route seems to develop earlier and remains unaffected by aging compared to the cognitive route. Moreover, state variables like the shared emotions' valence, the experienced affiliation with others or the motivation to take someone's perspective and personality traits like agreeableness selectively affect the intra- and interindividual capacity to empathize or to engage in ToM (see Table 1 for a summary).

Given this separability of the phenomena of affect sharing and perspective-taking, we argue for clear-cut terminology that differentiates among them. An argument for restraining the term empathy to affect sharing, as is being done in a large portion of the current literature (2, 3, 46, 47, 102, 103), is that it makes usage of the term unmistakable and distinctive. The umbrella usage, in contrast, requires specification as to which component is actually referred to in order to avoid misunderstanding. While a few studies also dissociate affective and cognitive components of ToM (104), the term ToM is used much more consistently already for what the umbrella usage would describe as cognitive empathy. Thus, there is no need for or reason to expand the term empathy to account for the phenomenon of perspective-taking. We believe clear-cut terminology is best suited to further research in the field (105, 106).

Foci of future research should be on (i) longitudinal developmental investigations, (ii) comprehensive assessments of empathy and ToM in psychopathology and subclinical variability as well as (iii) probing the differential plasticity of these social affective and cognitive capacities. Longitudinal studies could give in-depth understanding of the bases and influencing factors that affect the emergence and decline in empathy and perspective-taking. Differential development of the underlying brain structures could be informative regarding the differentiation of developmental empathy and ToM trajectories (107). Further research on situational, personality, and psychopathology factors related to empathy and ToM is needed to understand whether differences reflect alterations in the propensity or the capacity to mobilize these functions. Lastly, first evidence on the differential plasticity of social affect and cognition (108, 109) should be followed up with studies in clinical groups that show social interaction deficits.

## Author Contributions

All authors listed have made a substantial, direct and intellectual contribution to the work, and approved it for publication.

### Conflict of Interest Statement

SK is Specialty Chief Editor of Frontiers in Psychiatry–Social Cognition. The remaining authors declare that the research was conducted in the absence of any commercial or financial relationships that could be construed as a potential conflict of interest.
